# Family medicine in a globally-connected world: a South African perspective and the RCGP International and Overseas Network

**DOI:** 10.3399/bjgpopen17X101289

**Published:** 2017-11-15

**Authors:** David Rogers

**Affiliations:** 1 Senior Lecturer, Department of Family Medicine, University of Pretoria, Pretoria, South Africa; 2 Founder Member RCGP International and Overseas Network, RCGP, London, UK

**Keywords:** South Africa, general, practice, Royal College of General Practitioners, International and Overseas Network

The recent launch of the International and Overseas Network (ION) by the Royal College of General Practitioners (RCGP), presented a unique opportunity to review family medicine across the globe. This article looks at the future of family medicine from a South African perspective.

In 2007, family physicians were recognised for the first time as medical specialists in South Africa. Training is exclusively in the state sector, and predominantly hospital-based. Family physicians lead ward-based outreach teams composed mostly of community health workers and primary healthcare nurses.^1^ Unlike many British GPs, most South African family physicians also have other, more traditional hospital roles such as casualty, anaesthesia, or surgery duties. In 2014, to overcome the challenge of training to established international standards while retaining sensitivity to local needs, the South African Academy of Family Physicians (SAAFP) partnered with the RCGP to improve the training of family physicians in South Africa. The programme is called *Training the Trainers* and initial research indicates participants perceived a positive impact on the quality of training.^2^

To understand the broader impact of South African family physicians, it is also important to review statistics. In 2015, there were 545 family physicians or 0.1 per 10 000 population.^3^ In the UK in 2011, the equivalent statistic was just over 6 GPs per 10 000 population.^4^

The manpower problem in South Africa is exacerbated by unequal distribution of healthcare workers between private and state sectors, and rural and urban regions (Tables 1 and 2).^5^

**Table 1.** Distribution of healthcare workers and patients across private and public sector health care

	State hospital and clinics	Private hospitals and clinics
Registered medical practitioners, %	31	69
Population as patients, %	85	15

**Table 2.** Distribution of healthcare workers and patients across urban and rural regions

	Urban areas	Rural areas	
Registered medical practitioners, %	88	12	
Population as patients, %	57	43	

For primary healthcare teams to meet the healthcare needs of the whole South African population, it is clear that more needs to be done than just training small numbers of high quality family physicians.

To address these challenges in South Africa, a new type of health worker was created in 2008. Clinical associates (CAs) are mid-grade health workers who are trained over 3 years, predominantly by family physicians and mostly in rural clinical learning centres. CAs work in teams with doctors, taking on clinical roles for which they have been trained, and thus should reduce the workload of the existing family physicians. Early indications (Tables 3 and 4), suggest that the CA programme is achieving success in tackling the uneven distribution of healthcare workers.^6^

**Table 3.** Distribution of clinical associates across urban and rural regions

	Urban	Rural
Current work area, %	47	53
Future work intention, %	43	57

**Table 4.** Distribution of clinical associates by current employment

	State	Private	NGO	Military	Academic	Other
Current employer, %	60	8	12	13	7	7^a^

^a^Candidates were asked to tick all relevant boxes. NGO = non-governmental organisation.

Further research showing small but positive improvements in workload and cost-effectiveness is also emerging.^7,8^ Gains are currently small but international reports suggest these could increase as the number of CAs increase.^9^

Specialties such as emergency medicine, palliative care, and public health have opened further training opportunities for CAs, and many are now moving away from primary care settings, discouraged by the lack of further training opportunities in family medicine. If South African family physicians do not respond by addressing this lack of postgraduate training, the recent gains seen in rural clinics may soon be lost.

General practice in the UK is also facing a workload crisis, and here also the increased use of mid-grade health workers (pharmacists and physician associates) has begun. The *General Practice Forward View* proposes an extra 1000 physician associates, and 1500 pharmacists in primary care by 2020.^10^

In both countries, primary care teams of the future could have new members with new roles, but these roles require training and support. In both countries, as yet, there are very few opportunities for mid-grade health workers to further their careers in family medicine, and this could hamper existing efforts to alleviate the primary care workload crisis.

Extending the current partnership between the RCGP and SAAFP to offer quality postgraduate training opportunities for mid-grade health workers in family medicine could benefit both countries, but only if both bodies recognise the importance of supporting new types of family medicine practitioners.
